# Supplementary material for: Controlled Release of Poly(U) via Acetalated Dextran Microparticles for Enhanced Vaccine Adjuvant Delivery
Source: bioRxiv. 2025 Jul 21:2025.07.16.665134. Preprint. [Version 1] doi: 10.1101/2025.07.16.665134 (PMC12330755; doi:10.1101/2025.07.16.665134)
Supplement: Supplement 1 [file media-1.docx]

**SUPPLEMENTAL DATA: Controlled Release of Poly(U) via Acetalated Dextran Microparticles for Enhanced Vaccine Adjuvant Delivery**

Sophia A. Ly^1^, Nicole Rose Lukesh^2^, Erik S. Pena^1^, Ryan N. Woodring^2^, Sophie E. Mendell^2^, Connor T. Murphy ^2^, Grace L. Williamson ^2^, Kierstin A. Clark^2^, Alexandra M. Lopez^2^, Eric M. Bachelder^2^, Kristy M. Ainslie^1,2,3,*^

^1^Lampe Joint Department of Biomedical Engineering, NC State/UNC, Chapel Hill, NC, USA

^2^Division of Pharmacoengineering & Molecular Pharmaceutics, Eshelman School of Pharmacy, UNC, Chapel Hill, NC, USA

^3^ Department of Microbiology and Immunology, School of Medicine, UNC, Chapel Hill, NC, USA

*
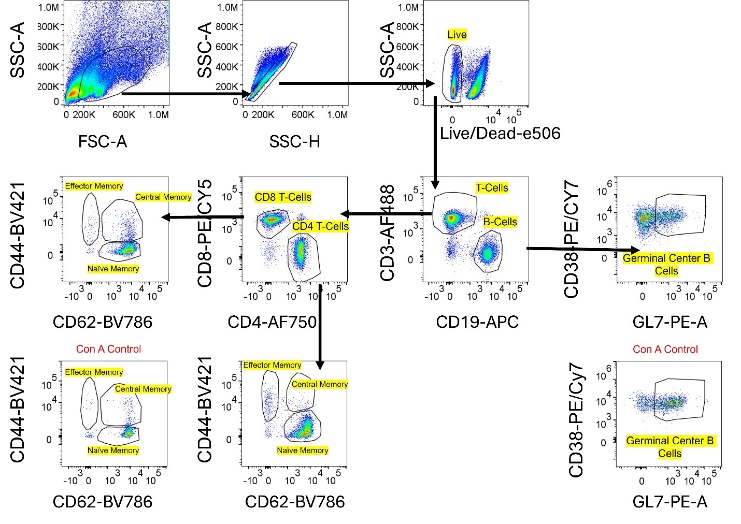
*

Supplemental Figure 1. Flow Gating Strategy for Splenocytes

*
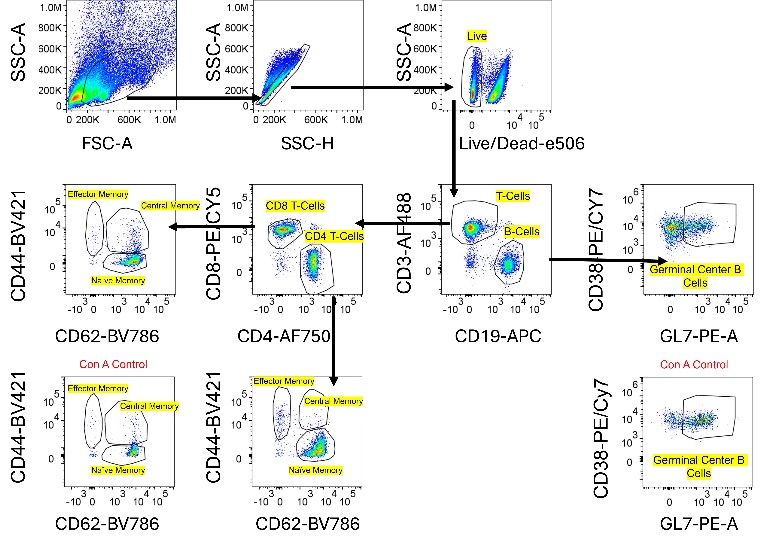
*

Supplemental Figure 2. Flow Gating Strategy for Lymphocytes

*
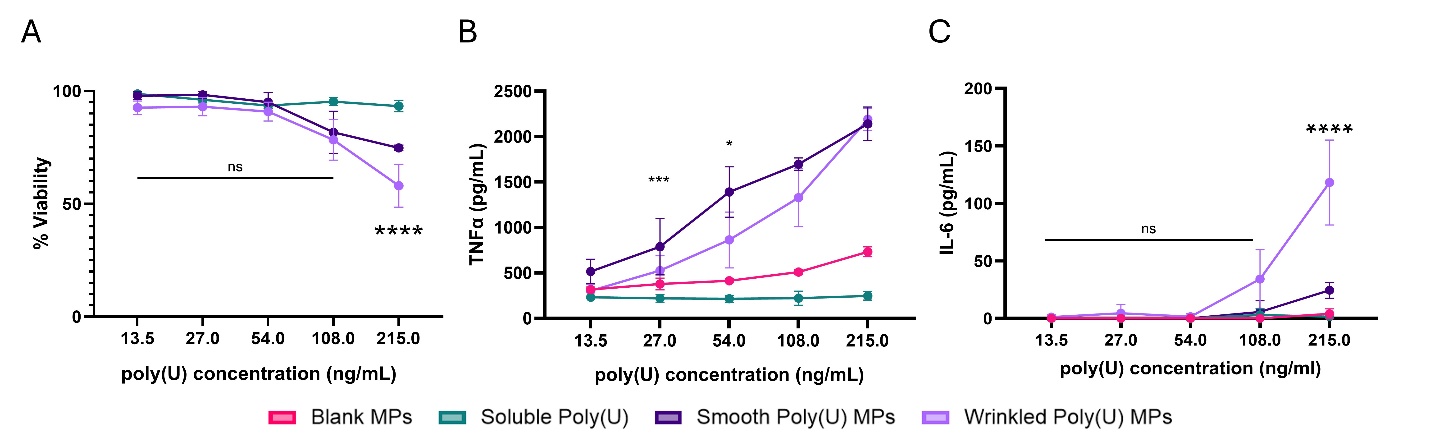
*

Supplemental Figure 3. Wrinkled poly(U) MPs had lower percent viability and higher IL-6 activation. DC2.4s were incubated with Wrinkled or smooth poly(U) MPs and soluble poly(U) for 24 hours. After 24 hours (A) cell viability was measured with Cell Titer Blue assay and (B) TNF-α IL-6 concentration in the culture supernatant was measured by cytokine ELISA. **** p ≤ 0.001.

*
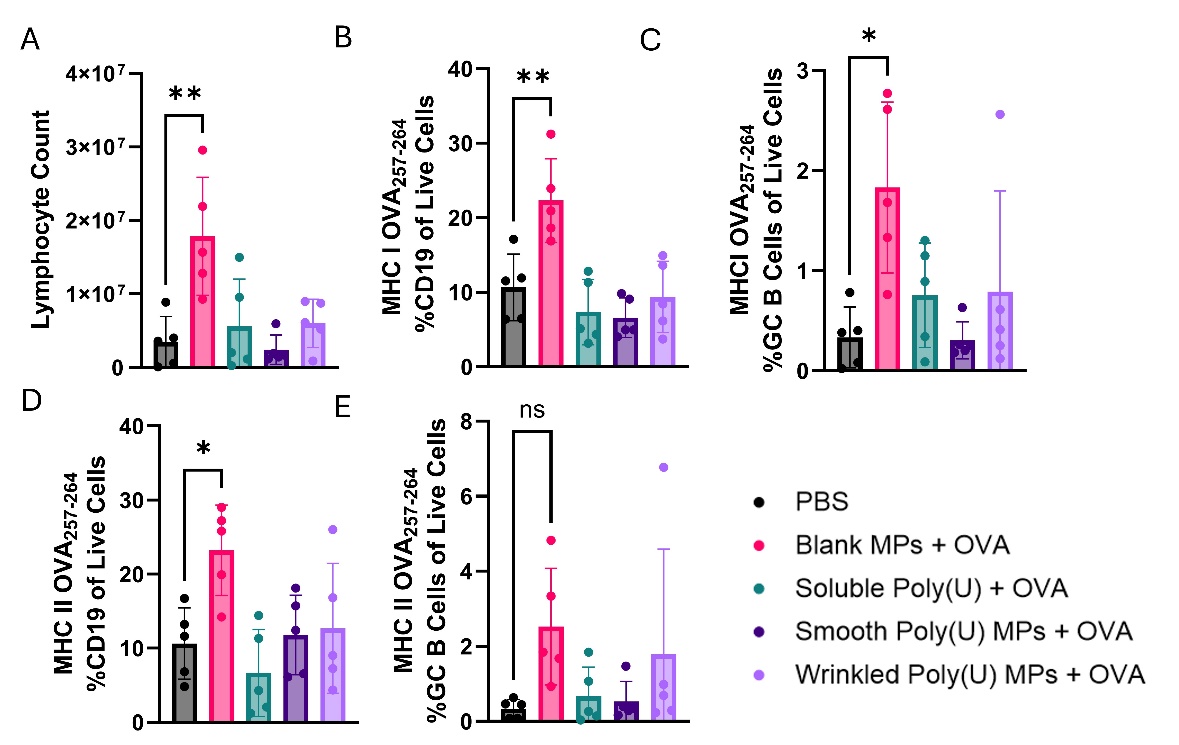
*

Supplemental Figure 4. After vaccination, Blank MPs with OVA bias cells towards a local B cell response. Using a prime-boost-boost vaccination schedule, inguinal lymph nodes (iLNs) were collected on day 45 and restimulated with full MHC-I and MHC-II peptide and run on flow cytometry. **(A)** Lymphocyte Count **(B)** percentage of CD19 in MHC-I restimulation, **(C)** Percentage of GC B cells in MHC-I restimulation, **(D)** percentage of CD19 in MHC-II restimulation and **(E)** percentage of germinal center (GC) B cells in MHC-II restimulation. Data presented as mean ± standard deviation. * p≤0.05 ** p≤0.01

*
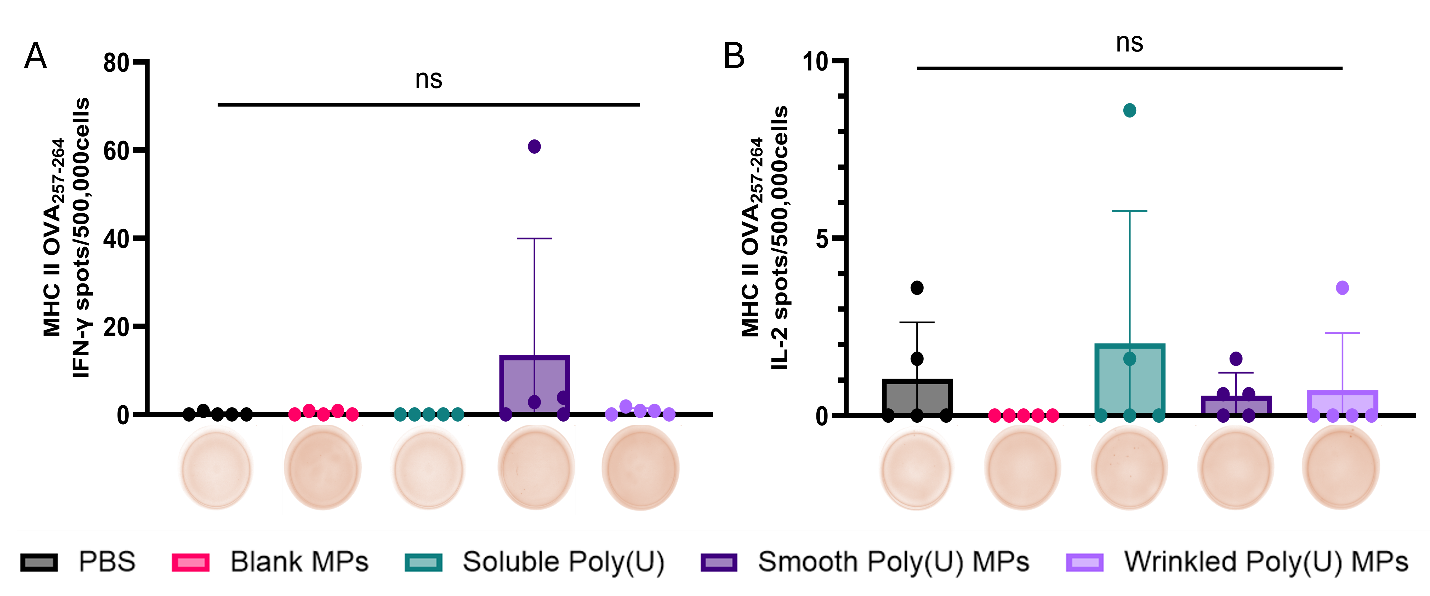
*

Supplemental Figure 5. No treatment groups have a strong recall immune response during antigen recall by splenocytes from day 42 vaccinated mice when restimulated with MHC-II. Spleens were isolated, made into single-cell suspensions to evaluate their cellular response on day 45 post prime + boost + boost vaccination, and stimulated and had no significant expression from ELISpot for (A) MHC-II and IFN-γ and (B) MHC-II peptide and IL-2. Representative images of an ELISpot well are shown under their corresponding vaccination group; data is from two-way ANOVA and shown as mean ± standard deviation.
